# Interferon gamma-inducible protein 16 in primary Sjögren’s syndrome: a novel player in disease pathogenesis?

**DOI:** 10.1186/s13075-015-0722-2

**Published:** 2015-08-14

**Authors:** Alessia Alunno, Valeria Caneparo, Francesco Carubbi, Onelia Bistoni, Sara Caterbi, Elena Bartoloni, Roberto Giacomelli, Marisa Gariglio, Santo Landolfo, Roberto Gerli

**Affiliations:** Rheumatology Unit, Department of Medicine, University of Perugia, Perugia, Italy; Virology Unit, Department of Translational Medicine, Novara Medical School, Novara, Italy; Rheumatology Unit, Department of Biotechnological and Applied Clinical Sciences, University of L’Aquila, L’Aquila, Italy; Viral Pathogenesis Unit, Department of Public Health and Pediatric Sciences, Turin Medical School, Turin, Italy

## Abstract

**Introduction:**

There is evidence that interferon is involved in the pathogenesis of primary Sjögren’s syndrome (pSS). The interferon-inducible IFI16 protein, normally expressed in cell nuclei, may be overexpressed, mislocalized in the cytoplasm and secreted in the extracellular milieu in several autoimmune disorders. This leads to tolerance breaking to this self-protein with consequent development of anti-IFI16 antibodies. The aim of this study was to identify the pathogenic and clinical significance of IFI16 and anti-IFI16 in pSS.

**Methods:**

IFI16 and anti-IFI16 were assessed in the serum of 67 pSS patients and over 100 healthy donors by enzyme-linked immunosorbent assay. IFI16 was also evaluated by immunohistochemistry in minor salivary glands of 15 pSS patients and 10 subjects with sicca symptoms but without any clinical, serological or histological features of pSS.

**Results:**

pSS patients display higher serum levels of both IFI16 and anti-IFI16 compared to healthy donors. IFI16 concentration was directly correlated with disease duration and focus score and inversely correlated with age at diagnosis. Moreover, IFI16 positivity was associated with concurrent positivity for rheumatoid factor. Interestingly, the direct correlation between IFI16 positivity and focus score was independent of disease duration and age at diagnosis. pSS minor salivary glands display marked expression and cytoplasmic mislocalization of IFI16 by acinar and ductal epithelial cells as well as infiltrating lymphocytes and peri/intralesional endothelium compared to minor salivary glands with normal architecture or nonspecific chronic sialadenitis. Within the mononuclear cell infiltrate, IFI16 expression appears to parallel the distribution of T lymphocytes.

**Conclusion:**

Our data suggest that the IFI16 protein may be involved in the pathogenesis of glandular inflammation occurring in pSS.

## Introduction

Primary Sjögren’s syndrome (pSS) is a chronic systemic autoimmune disease that primarily affects exocrine glands. Clinical presentation considerably varies from relatively mild sicca symptoms to severe systemic involvement, with an increased risk of developing non-Hodgkin’s lymphoma [1]. Classically, minor salivary glands (MSGs) of patients with pSS show a focal lymphocytic sialadenitis (FLS) characterized by the presence of lymphocyte aggregates usually located in perivascular or periductal areas. The spectrum of MSG histopathological damage ranges from mild to diffuse infiltrates with progressive loss of normal glandular tissue. T cells predominate in mild lesions, whereas B cells are the most represented cell subset in the advanced lesions. The infiltrating lymphocytes are often organized into tertiary lymphoid tissues in nonlymphoid locations, also known as ectopic lymphoid structures, showing a network including specific segregated T- and B-cell zones and follicular dendritic cells. Some of these tertiary lymphoid tissues are arranged in germinal centers (GCs) [2]. To date, the pathogenesis of pSS is not fully elucidated. However, a growing number of studies suggest that the so-called interferon (IFN) signature is involved in the induction and perpetuation of the disease [3, 4]. In particular, increased production of IFNs and marked upregulation of type I IFN-regulated gene transcripts, described in pSS patients as well as in animal models of the disease, suggest a strong response due to glandular apoptosis and toll-like receptor activation [5–7]. In this context, the interferon gamma-inducible protein 16 (IFI16), a member of the HIN200/Ifi200 family of IFN-inducible genes, has been drumming up growing interest for its possible role in initiation and progression of chronic inflammatory autoimmune disorders. IFI16 is constitutively expressed in the nucleus of hematopoietic cells, particularly lymphocytes, vascular endothelial cells and keratinocytes [8]. Although it is a multifaceted protein involved in cell cycle regulation, tumor suppression, endothelial cell apoptosis and DNA damage signaling [9–12], great interest is derived from the evidence that its overexpression may drive early steps of an inflammatory response through nuclear factor-kB-mediated secretion of pro-inflammatory molecules. In this context, it is important to note that IFI16, normally expressed in cell nuclei, may be overexpressed in several autoimmune disorders. Its overexpression, cytoplasmic mislocalization and extracellular appearance during cell death lead to the breaking of tolerance to this self-protein with consequent development of anti-IFI16 antibodies [13]. Therefore, the release of IFI16 in the extracellular milieu may mark the first step in the development of autoimmunity [14]. The presence of significant levels of extracellular IFI16 protein and anti-IFI16 antibodies have been recently identified in the sera of patients affected by different systemic autoimmune diseases, including pSS [10, 12, 15–17], thereby confirming its presence in the extracellular milieu and its possible role as an autoantigen [14]. Taken together, these data provide evidence for a novel alarmin function of IFI16 protein which can be overexpressed upon inflammatory stimuli and released in the extracellular environment with eventual endothelial cell binding causing tissue damage. However, a number of questions regarding the role of IF16 are still open. Thus, we were prompted to analyze possible pathogenic, diagnostic and prognostic significance of IFI16 protein and anti-IFI16 antibodies in patients with pSS, a combined model of systemic autoimmune and chronic inflammatory disorder.

## Methods

### Patients

Sixty-seven consecutive female patients with pSS, classified according to the European–American criteria [18], with a mean age of 59 years (standard error of the mean (SEM) = 1.5) were enrolled. At the time of enrollment, serum samples were collected and stored at −20 °C until use and clinical and serological features were recorded. Concerning hematological abnormalities, leukopenia was defined as ≤3000 leukocytes/mL, hypergammaglobulinemia was defined as an IgG level over 16 g/L and hypocomplementemia was defined by a reduction of C4 or C3. One-hundred and eighty two healthy blood donors, 145 females and 37 males, with a mean age of 51 years (SEM = 1.2) acted as controls (healthy donors; HDs). Fifteen pSS MSGs, collected at the time of diagnosis, were also retrospectively evaluated. Ten MSGs obtained from subjects with sicca symptoms, but without any clinical and serological features of pSS, acted as controls (five normal MSGs and five showing different grades of nonspecific chronic sialadenitis (NSCS), without FLS). The whole study was approved by the local Ethics Committee (Comitato Etico delle Aziende Sanitarie dell’Umbria, CEAS) and written informed consent was obtained in accordance with the declaration of Helsinki.

### Determination of extracellular IFI16 protein by capture ELISA

A capture enzyme-linked immunosorbent assay (ELISA) was employed for determination of circulating extracellular IFI16 protein following a procedure detailed elsewhere [15]. Briefly, polystyrene micro-well plates (Nunc-Immuno MaxiSorp; Nunc, Roskilde, Denmark) were coated with a home-made polyclonal rabbit-anti-IFI16 antibody (aa 478–729). Subsequently, the plates were washed and free binding sites were saturated with phosphate-buffered saline (PBS)/0.05 % Tween/3 % bovine serum albumin (BSA). After blocking, sera were added in duplicate. Purified 6His-IFI16 protein was used as standard. BSA served as negative control. The samples were washed and in each case monoclonal mouse anti-IFI16 antibody (Santa Cruz, sc-8023) was added and incubated for 1 h at room temperature. After washing, horseradish peroxidase-conjugated anti-mouse antibody (GE Healthcare Europe GmbH, Milan, Italy) was added. Following the addition of the substrate (TMB; KPL, Gaithersburg, MD, USA), absorbance was measured at 450 nm using a microplate reader (SpectraCount, Packard, Packard BioScience Company). The determination of the concentration was carried out using a standard curve for which increasing concentrations of purified 6His-IFI16 were used.

### Determination of antibody titers towards human recombinant IFI16 by ELISA

To detect anti-IFI16 antibodies, polystyrene micro-well plates (Nunc-Immuno MaxiSorp; Nunc) were coated with a solution of recombinant IFI16 in PBS and, after blocking, sera were added in duplicate. After washing, horseradish peroxidase-conjugated rabbit anti-human IgG (Dako Cytomation, Carpinteria, CA, USA) was added. Following the addition of the substrate (TMB; KPL), absorbance was measured at 450 nm using a microplate reader (SpectraCount, Packard). The background reactivity of the reference mixture was subtracted to calculate the results. A standard curve was constructed by serially diluting IgG from an anti-IFI16-positive patient serum [16, 17].

### Histological analysis of MSG biopsies

Labial MSG specimens were obtained from 15 pSS patients and 10 HDs according to international guidelines. All sections were randomly analyzed by two expert observers, blinded to clinical and laboratory data. Each sample was independently evaluated and any discrepancies were resolved by consensus. The histological pattern (normal, NSCS, FLS) was assessed using hematoxylin-eosin stained sections [19, 20]. FLS pattern, which is diagnostic for pSS, is characterized by the presence of at least one focus, namely an aggregate of at least 50 lymphocytes, in the glandular tissue. NSCS pattern is characterized by scattered lymphocyte aggregates that do not reach the number of 50 cells and therefore cannot be classified as foci.

Both cellular infiltrate and lymphoid organization, including the presence of GC-like structures, were assessed by immunofluorescence staining of sequential sections with monoclonal antibodies recognizing CD3, CD20 and CD21 (all provided by DakoCytomation, Glostrup, Denmark), as previously described [2, 21].

IFI16 expression was evaluated using standard immunohistochemistry, as already described in a previous study [13]. Images were acquired using an Olympus BX53 fluorescence microscope with CellSens software (Olympus America Inc., Center Valley, PA, USA).

### Statistical analysis

Data analysis was performed using IBM-SPSS version 13.0 (IBM, Armonk, NY, USA). Mann Whitney *U* test or Chi square test were employed to compare variables among subgroups. Binary logistic regression was used to identify possible association between clinical and serological features and the presence of serum IFI16 protein and anti-IFI16 antibody. The significance level was two sided and set at *p* < 0.05.

## Results

### Serum IFI16 protein and anti-IFI16 antibody detection

**Fig. 1:**
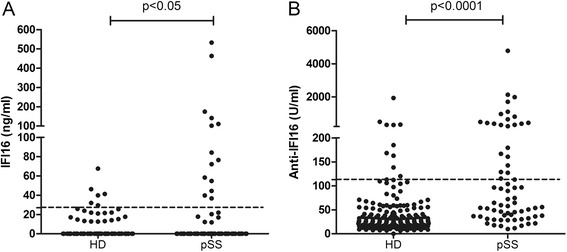
Expression of interferon gamma-inducible (IFI16) protein (**a**) and anti-IFI16 antibodies (**b**) in healthy donors (HD; n = 116 and n = 182, respectively) and primary Sjögren’s syndrome (pSS) patients (n = 67). Bars indicate mean values. Dotted lines mark cut-off levels of positivity determined as the 95th percentile of the control population. *p* values were calculated with Mann Whitney *U* test

As shown in Fig. 1, serum levels of both IFI16 protein and anti-IFI16 antibodies were higher in pSS than in HDs (*p* < 0.05 and *p* < 0.0001, respectively). According to a cut-off established as the 95th percentile of the control population, positivity for IFI16 protein and for anti-IFI16 was considered for values ≥27 ng/ml and ≥113 U/ml, respectively. IFI16 was significantly more prevalent in pSS compared to HDs as it was detectable in 14/67 patients (21 %) and in 6/116 HDs (5 %) (*p* < 0.0001). Similarly, a significantly higher prevalence in pSS was confirmed also for anti-IFI16 antibodies that were present in 23/67 pSS patients (34 %) and in 9/182 HDs (5 %) (*p* < 0.0001).

### Clinical and laboratory associations with serum IFI16 protein and anti-IFI16 antibodies

Table 1 summarizes clinical and serological features in the overall pSS patient population and in patients subdivided according to the presence or absence of serum IFI16/anti-IFI16. We failed to observe any differences among subgroups except for a higher prevalence of rheumatoid factor (RF) in IFI16^+^ patients. We subsequently evaluated whether IFI16 serum concentration, rather than its presence, could be correlated to any clinical variable. A direct correlation with disease duration (Spearman’s rho = 0.32; *p* = 0.008) and an inverse correlation with age at diagnosis (Spearman’s rho = 0.24; *p* = 0.04), without any association with patients’ age, were observed (data not shown). Interestingly, all IFI16^+^ patients displayed a disease duration >3 years. No association between IFI16 positivity or concentration and disease activity, calculated with the EULAR Sjögren’s syndrome disease activity index, was observed. Finally, no correlations between anti-IFI16 antibody positivity or titer and any clinical and/or serological parameters were identified.

**Table 1:** Demographic, clinical and serological characteristics of pSS patients

	All pSS	IFI16 neg	IFI16 pos	*p*	anti-IFI16 neg	anti-IFI16 pos	*p*
Patient number (n)	67	53	14	–	44	23	–
Age (years)^a^	59 ± 1	60 ± 2	55 ± 3	0.2	60 ± 2	56 ± 2	0.2
Age at diagnosis (years)^a^	49 ± 2	50 ± 2	45 ± 3	0.3	51 ± 2	46 ± 2	0.3
Disease duration (years)^a^	9 ± 1	9 ± 1	10 ± 1	0.1	9 ± 1	10 ± 2	0.1
Xerophtalmia	92.5	94.3	85.7	0.3	86.4	82.6	0.7
Xerostomia	85.1	88.7	71.4	0.2	90.9	95.7	0.6
Salivary gland swelling	47.8	49.1	42.9	0.8	50.0	43.5	0.8
Extraglandular manifestations^b^	76.1	77.3	71.4	0.7	79.5	69.5	0.4
Leukopenia	37.3	37.7	35.7	0.9	38.6	34.8	0.8
Hypergammaglobulinemia	61.2	56.6	78.6	0.2	61.4	60.9	0.8
Hypocomplementemia	26.9	22.6	42.9	0.2	25.0	30.4	0.7
Autoantibodies							
Neither anti-SSA nor anti-SSB	16.4	17.0	14.3	0.9	18.2	13	0.7
Anti-SSA only	26.9	28.3	21.4	0.7	20.5	39.1	0.1
Anti-SSA and anti-SSB	56.7	54.7	64.3	0.6	61.4	47.8	0.3
RF ± anti-SSA ± anti-SSB	68.7	60.4	100	0.003	70.5	65.2	0.8

^a^Values are reported as mean ± standard error of the mean and corresponding *p* values are calculated with Mann Whitney *U* test. All other values are reported as percentage of patients and corresponding *p* values are calculated with chi square test. ^b^Percentage of patients with at least one extraglandular manifestation including articular, pulmonary or esophageal involvement, purpura, Raynaud’s phenomenon and lymphadenopathy. *IFI16* interferon gamma-inducible protein 16, *neg* negative, *pos* positive, *pSS* primary Sjögren’s syndrome, *RF* rheumatoid factor

### Histological evaluation of IFI16 protein at glandular level

**Fig. 2:**
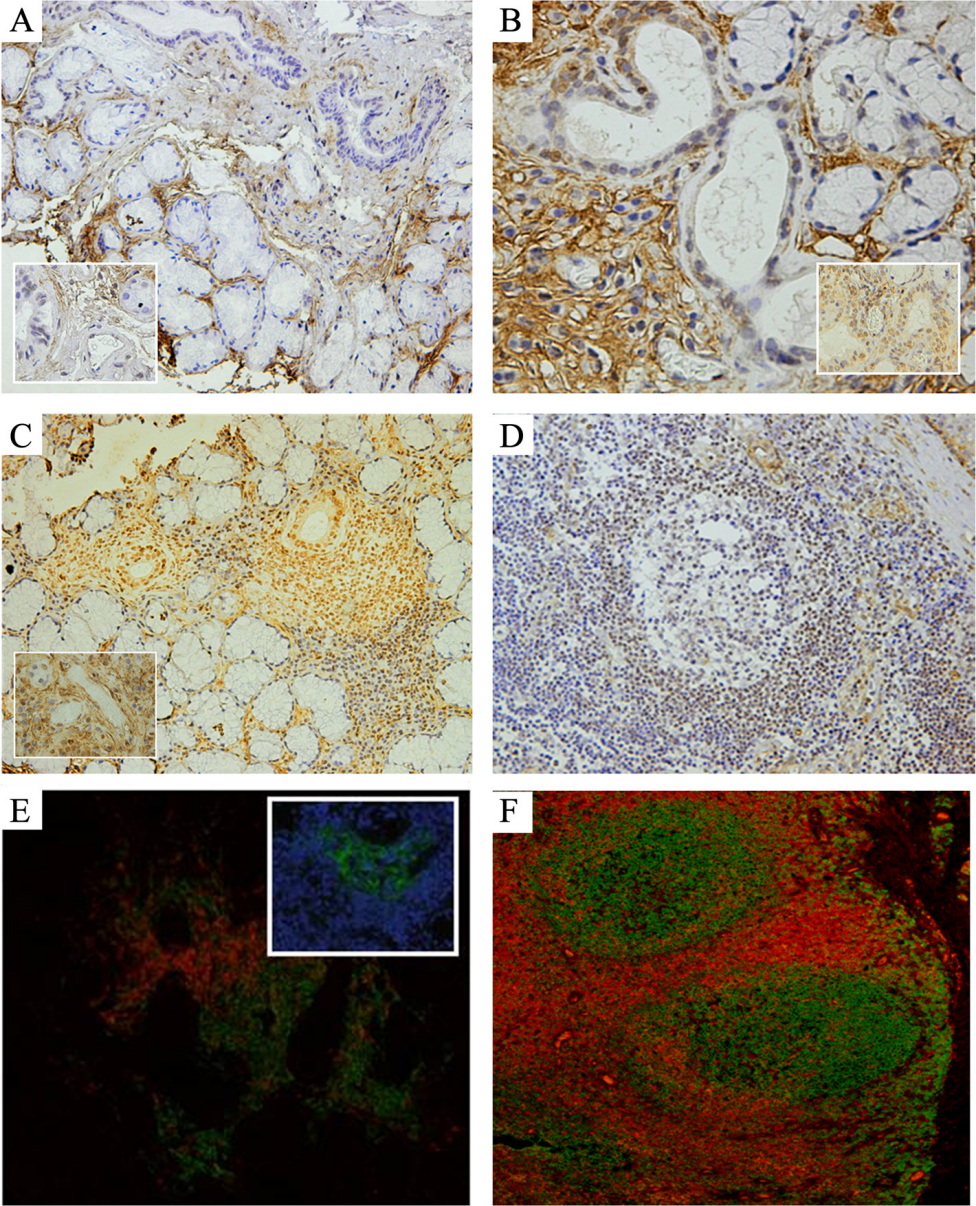
Expression of IFI16 in MSG and tonsil. Immunohistochemical analysis for IFI16 in MSG with normal architecture (**a**), NSCS (**b**), FLS (**c**) and in tonsil (**d**). Inserts in **a**, **b** and **c** depict a detail of endothelial cells from the corresponding panel. **e**, **f** Double immunofluorescence staining for CD3 (red) and CD20 (green) in FLS (**e**) and tonsil (**f**). Insert in **e** depicts immunofluorescence staining for CD21 (green) and DAPI (blue), representing a GC-like structure in the CD20^+^ B-cell area of panel **e**

The histological analysis of normal MSGs revealed that IFI16 protein was not constitutively expressed by glandular tissue (Fig. 2a). Conversely, few nuclei of glandular epithelial cells were positive for IFI16 staining in NSCS. A cytoplasmic localization of this protein could be only occasionally detected in glandular epithelial cells (Fig. 2b). In this context, IFI16 was also displayed by the nuclei of inflammatory mononuclear cells (Fig. 2b). The scattered or small aggregates of plasma cells, normally present in MSGs or during NSCS, appeared to have nuclei with a lower IFI16 positivity when compared with other inflammatory cells. In contrast, in pSS biopsies intense IFI16-positive nuclei were found in both ductal and acinar epithelial cells. A stronger cytoplasmic staining with respect to normal and NSCS was detected in ductal epithelial cells, while the detection of IFI16 staining in the cytoplasm of acinar epithelial cells was hampered by abundant secretion products (Fig. 2a–c). IFI16 nuclear and cytoplasmic staining was also observed in the inflammatory cells involved in FLS (Fig. 2c). A more detailed analysis of inflammatory infiltrate, according to the distribution of T and B lymphocytes and the presence of GCs, showed a predominance of IFI16 staining in the T- rather than B-cell area containing GC-like structures (Fig. 2e). This particular IFI16 distribution, paralleling B/T cell segregation, was also detectable in the inflammatory follicles of tonsil specimens (Fig. 2d and f). Concerning the nuclear staining for IFI16 in endothelial cells, our results showed that the constitutional expression of IFI16 was more pronounced in pSS FLS peri- and intralesional endothelium compared to normal and NSCS patterns (Fig. 2a–c, inserts).

### Correlations between IFI16 protein serum levels and glandular infiltration degree

Taking the aforementioned observations in the serum and in MSGs, we attempted to identify a possible association between circulating IFI16 and histological features. The median focus score (FS), namely the number of foci in 4 mm^2^ of glandular tissue, was 3.5 (range 0–12). We observed that IFI16 concentration was directly correlated with the FS at the time of diagnosis (Spearman’s rho = 0.30; *p* = 0.02). Furthermore, this association was quantified by binary logistic regression that revealed an odds ratio of 1.4 (95 % confidence interval 1.03 to 1.9; *p* = 0.03) for the FS. However, since also disease duration and age at diagnosis were found to be correlated with IFI16, we corrected the analysis according to these variables and observed that FS was associated with serum IFI16 independently off disease duration and age at diagnosis (odds ratio = 1.5; 95 % confidence interval 1.02 to 1.9; *p* = 0.04).

## Discussion

Several data from animal models and patients highlighted the pathogenic role of the IFN signature in pSS [6, 7], thereby supporting the evaluation of the IFN-inducible gene role as an intriguing issue. In the present study, we confirmed that patients with pSS display circulating IFI16 as well as antibodies reactive against this protein. More interestingly, however, our data appear to suggest that the IFI16 protein may be involved in the development of glandular inflammation occurring in pSS. In fact, unlike normal and NSCS MSGs, a marked expression of this molecule by acinar and ductal epithelial cells as well as infiltrating lymphocytes and peri/intralesional endothelium was found in pSS MSGs.

In recent years, a better understanding of pSS pathogenesis has allowed the identification of salivary gland epithelial cells (SGECs) as active players rather than innocent bystanders in the initiation and perpetuation of the disease [22]. In particular, following IFN ligation to toll-like receptors (TLRs), such as TLR3, SGECs upregulate and mislocalize nuclear proteins in the cytoplasm that are normally segregated from the immune system. These autoantigens, including Ro52 and Ro60, are subsequently released in the extracellular milieu either within exosomes or following SGEC apoptosis [23]. The consequence of these events trigger activation of T lymphocytes and an antigen-specific B-cell recruitment leading to the production of autoantibodies, such as anti-Ro52 and anti-Ro60, key elements of pSS classification criteria [18].

This scenario appears to fit with our findings concerning IFI16 at the glandular level, where the predominant chronic inflammatory process takes place, and anti-IFI16 in the bloodstream. Therefore, it is reasonable to speculate that IFI16 may undergo the same fate as the aforementioned Ro52 and Ro60 nuclear proteins in SGECs. Of interest, in addition, similarly to anti-Ro52 and anti-Ro60 autoantibodies, those against IFI16 are also displayed by some, but not all, pSS patients.

Finally, it is worth mentioning that while some epithelial tissues, including skin, gastrointestinal tract, urogenital tract, and glands and ducts of breast tissues constitutively express IFI16 [8], normal MSGECs do not display the IFI16 protein. This observation underscores that IFI16 overexpression observed in NSCS and, to a greater extent, in pSS results from a “de novo” synthesis of such a protein. The presence of free IFI16 protein in the serum could reflect cell secretion of the protein in the extracellular milieu. The fact that we were able to detect the protein in the serum from only a subgroup of patients is not so surprising, since it is conceivable that the main pathogenic role of IFI16 is exerted at the glandular tissue level. Indeed, autoreactive B lymphocytes in pSS are generated in target tissue where autoantigen release by SGECs is massive. Only when the inflammatory infiltrate is particularly severe can the protein be released in larger amounts in the circulation, as supported by our finding showing a direct correlation between level of serum IFI16 and degree of MSG inflammatory score.

In this setting, our observations that circulating IFI16 levels were associated with RF and inversely correlated with age at disease diagnosis are particularly intriguing. Indeed, although we failed to identify any association with disease activity, this finding appears to fit with the observation that patients diagnosed at a younger age are more likely to have circulating autoantibodies and usually display more severe glandular inflammation [2, 24, 25].

## Conclusion

In conclusion, although we are aware that our study displays some limitations due to its retrospective nature and the low number of patients, we believe that our results may shed some light on the role of IFN-inducible genes in pSS pathogenesis. Larger and prospective studies, however, are required to point out a possible application of serum IFI16 protein detection in the current clinical practice.

## Abbreviations

BSA: Bovine serum albumin
ELISA: Enzyme-linked immunosorbent assay
FLS: Focal lymphocytic sialadenitis
FS: Focus score
GC: Germinal center
HD: Healthy donor
IFI16: Interferon gamma-inducible protein 16
IFN: Interferon
MSG: Minor salivary gland
NSCS: Nonspecific chronic sialadenitis
PBS: Phosphate-buffered saline
pSS: Primary Sjögren’s syndrome
RF: Rheumatoid factor
SEM: Standard error of the mean
SGEC: Salivary gland epithelial cell
TLR: Toll like receptor
